# Circular RNA hsa_circ_0004543 Aggravates Cervical Cancer Development by Sponging MicroRNA hsa-miR-217 to Upregulate Hypoxia-Inducible Factor

**DOI:** 10.1155/2022/4031403

**Published:** 2022-03-09

**Authors:** Jun Liu, Yingqiao Liu, Yang Zhang, Jing Zheng, Shuzhen Wang, Guangming Cao

**Affiliations:** ^1^Department of Obstetrics and Gynecology, Beijing Chao-Yang Hospital, Capital Medical University, Beijing, China; ^2^Department of Obstetrics and Gynecology, Lu-He Teaching Hospital, Capital Medical University, Beijing, China

## Abstract

Cervical cancer (CC) is the 4^th^ principal source of cancer death in females with 604,000 new patients and 342,000 deaths in 2020 worldwide. It has been extensively shown that circRNAs are involved in regulating CC development. Nevertheless, the function and mechanisms of hsa_circ_0004543 in regulating CC need to be clearly elucidated. Herein, hsa_circ_0004543 expressions were compared between 40 paired paracancerous and cancerous specimens from CC patients and between 6 CC cell lines and a normal human cervical epithelial cell line based on qRT-PCR. Potential complementary binding sites between hsa-miR-217 and hsa_circ_0004543 were predicted using the interactome, while binding sites for the hypoxia-inducible factor-1a (HIF-1a) were predicted by TargetScan. The function and mechanism of hsa_circ_0004543 in the development of CC were estimated by silencing hsa_circ_0004543 with/without hsa-miR-217 or HIF-1a overexpression. The association between gene expressions was evaluated with Pearson's correlation analysis. Molecular mechanisms were explored by ribonucleic acid (RNA) pulldown, dual-luciferase activity, and rescue experimental assays. Our results revealed that the hsa_circ_0004543 expression was considerably increased in CC tissues and cells. Its silencing repressed proliferation and metastasis, while it increased apoptosis of CC cells. The investigation of the mechanism showed that hsa-miR-217 silencing or HIF-1a overexpression rescued hsa_circ_0004543, and silencing inhibited malignant phenotypes of CC cells. hsa_circ_0004543 upregulated the HIF-1*α* expression by sponging hsa-miR-217 in CC development. Therefore, the hsa_circ_0004543 functioned as a competing endogenous RNA (ceRNA) of hsa-miR-217 to increase CC oncogenesis and metastasis by the upregulation of the HIF-1*α* expression. Consequently, targeting the hsa_circ_0004543/hsa-miR-217/HIF-1*α* axis might be a potential treatment approach for CC.

## 1. Introduction

Cancer is now commonly acknowledged as a worldwide hazard to international development [1]. The latest United Nations high-level meeting on noncommunicable diseases (NCDs) confirmed this statement and further emphasized the slow development in meeting the 2011 Political Declaration on NCD prevention and control [1]. The lack of sufficient molecular mechanisms to detect new biomarkers for early diagnosis, antidrug development, and clinical outcome prognosis has been recognized as the main problems in reaching these goals. As a result, it is of great significance to discover novel biomarkers in cancers.

As the 4^th^ most commonly diagnosed malignancy, cervical cancer (CC) is the 4th principal source of female cancer deaths, with 604,000 new patients and 342,000 deaths in 2020 worldwide [2, 3].

CC is commonly asymptomatic and may be diagnosed during pelvic examination or routine screening in the early stages, with the symptoms of abnormal or postcoital vaginal bleeding [4]. The presence of large amounts of smelly vaginal discharge may also be a symptom [4, 5]. The triad of flank pain, lower-limb edema, and sciatica indicates pelvic sidewall invasion. The vaginal passage of urine is a bladder invasion symptom of vesicovaginal fistula, whereas the vaginal passage of feces is a rectum invasion symptom of rectovaginal fistula. Race, age, histological type, grade, stage, location, lymph-node involvement, treatment status, and tumor volume are all the prognostic factors for locally advanced cervical cancers [4–6]. Surgery combined with chemoradiation or chemotherapy is the main management of CC in its early stages.

Despite the fact that diagnostic and therapeutic advances of surgical treatment with concurrent chemoradiotherapy have improved the overall five-year survival to about 70% in advanced CC patients, the metastasis and recurrence of CC still result in a quite poor prognosis with a 5-year overall survival <30% in the majority of regions and countries owing to restricted clinical strategies [3]. Furthermore, almost 30% of patients die from disease relapse or development. Hence, to explore the molecular mechanisms involved in the development and progression of CC [7].

As a newly identified noncoding RNA (ncRNA) class, circular RNAs (circRNAs) are preserved throughout species and are more stable than linear RNAs [8–10]. Increasing evidence implies that circRNAs have the crosstalk capacity with RNA binding proteins to act as sponges of miRNA for regulating downstream gene expression [11]. circRNAs have been reported to be related to CC progression through various mechanisms, where the most important one is serving as a microRNA (miRNA) sponge [12–15]. Consequently, it is essential to find out the abnormal circRNAs and the involved new molecular mechanisms to develop the therapeutic targets for CC management.

hsa_circ_0004543 was significantly increased in CC patient tissues based on circRNA microarray analysis [12]. However, the specific role and related molecular mechanism of hsa_circ_0004543 in CC oncogenesis and metastasis need to be explored further.

Hypoxia-inducible factor-1 *α* (HIF-1*α*) is a key responser adapted to cancer hypoxia. HIF-1*α* signaling activated in hypoxia conditions contributes to cell biology associated with oncogenesis, a key issue restraining the chemotherapy efficiency in various cancer treatment including CC [16, 17]. As a main property of CC [18, 19], hypoxia regulates all steps of cancer metastasis from the initial step to eventually colonizing the target organs [20, 21]. Increased intratumor hypoxia stabilizes and activates HIF-1*α*, which may activate many metastatic sequences to promote local and distant site cancer recurrence [22, 23]. As a result, targeting hypoxia via diverse methods may decrease extensive cancer-intrinsic metastasis [21, 24].

Moreover, miR-217/HIF-1*α*/AXL signaling has been reported to be involved in lncRNA-HOTAIR-promoted renal cell carcinoma carcinogenesis, which provides a new target for the diagnosis and treatment of renal cell carcinoma [25].

Therefore, we speculated that hsa_circ_0004543 may stimulate CC development by hsa-miR-217/HIF-1*α* signaling. Herein, we intend to explore the function and molecular mechanisms of hsa_circ_0004543 in CC oncogenesis and development, thus providing a potential biomarker for better management of CC. After analyzing the expressions of hsa_circ_0004543, hsa-miR-217, and HIF-1*α* in 40 paired CC and paracancerous tissues with qRT-PCR, which revealed that hsa_circ_0004543 and HIF-1*α* were increased, while hsa-miR-217 was decreased in tissues of CC patients; hsa_circ_0004543 was further found to increase HIF-1*α* expression via sponging hsa-miR-217. Thus, this promoted CC oncogenesis and development. These findings may enable the progress of clinical management strategies against CC.

## 2. Materials and Methods

### 2.1. Reagents

The following reagents and instruments were used in this study: dual-luciferase reporter assay system (Promega Madison, US), fetal bovine serum (FBS), Dulbecco's modified eagle medium (DMEM) cell culture medium (Gibco, Rockford, US), radio immunoprecipitation assay (RIPA) buffer (Beyotime, Shanghai, China), Matrigel (BD, New Jersey, US), cell counting kit-8 (CCK8) assay kit (Dojindo Corp, Kyushu, Japan), Gene Mutation Kit and SYBR Green Premix Ex Taq™ II (TaKaRa, Dalian, China), Pierce^TM^ Magnetic RNA-Protein Pull-Down kit, Lipofectamine 3000, M-MLV reverse transcriptase kit, miRNA reverse transcriptase kit, TRIzol reagent, and SuperSignal West Dura Extended Duration Substrate (Thermo Fisher Scientific, Inc., Waltham, US). Antibodies were purchased from Santa Cruz (Dallas, US). Propidium iodide and APC-Annexin V were purchased form Sigma-Aldrich (St. Louis, USA). The PsiCHECK™-2 vector was purchased from Promega (Madison, US).

### 2.2. Patient Specimens, Consents, and Ethics

We collected the cancerous and paracancerous specimens from 40 CC patients during surgical treatment and stored them at -80°C. All patients provided written informed consent. All experimental procedures were approved by the Ethics Committee of the Beijing Chao-Yang Hospital at Capital Medical University.

### 2.3. Cells and Culture

The Committee on Type Culture Collection of the Chinese Academy of Sciences (Shanghai, China) provided all CC and normal human cervical epithelial (End1/E6E7) cells, which were routinely cultured in DMEM medium containing FBS (10%), penicillin (100 IU/mL), and streptomycin (100 mg/mL) [26].

### 2.4. Cell Viability Analysis

Cell viability was evaluated using the CCK-8 kit following the manufacturer's guidelines. In brief, 10 *μ*L of CCK-8 reagent was applied to each well of cells cultured in a 96-well plate with an original 2000 cells/well and incubated in the dark for 2 h at 37°C. The optional density (OD) value was determined at a wavelength of 450 nm with a microplate reader (Bio-Rad, Hercules) [27].

### 2.5. Colony Proliferation Evaluation

Cells were seeded in 6 well plates with 1000 cells/well and incubated for 10 days at 37°C to form colonies, followed by fixation in 4% paraformaldehyde for 10 min and staining in 0.5% crystal violet for 5 min. Colony numbers were determined using the software ImageJ (National Institutes of Health, Bethesda, MD, USA) and images were acquired with a light microscope (Olympus, Japan) [27].

### 2.6. Apoptosis Evaluation

Cells washed with precold phosphate buffer solution were fixed and incubated for 15 min with propidium iodide and APC-Annexin V, respectively, in dark conditions at room temperature. Apoptotic cells were then detected using a BD FACSCalibur flow cytometer. Apoptotic rates were determined using Cell Quest software (BDIS, San Jose, USA) [27].

### 2.7. Invasion and Migration Evaluation

A transwell (24-well, Corning Costar) was applied to evaluate cell invasion (with Matrigel coating) and migration (without Matrigel coating) abilities. In brief, 600 *μ*L of 10% FBS-supplied culture medium was loaded in the lower chamber, and 3 × 10^5^ cells in 200 *μ*l of serum-free medium were loaded in the upper chamber. After being cultured for 24 h (migration assay) or 48 h (invasion assay), the commonly used time points for cell migration and invasion assays to avoid the influence of cell proliferation, the migrated or invaded cells were fixed and stained [27].

### 2.8. Potential Binding Gene Prediction

Potential hsa_circ_0004543 sponged miRNAs were predicted with the online tool Circular RNA Interactome (https://circinteractome.nia.nih.gov/) [28]. Potential mRNAs binding to hsa-miR-217 were predicted with the TargetScan online tool (http://www.targetscan.org/) [29].

### 2.9. qRT-PCR Assay

Sangon (Shanghai, China) synthesized the primers. Cells were lysed in Trizol to isolate the total RNA according to the protocols. RNAs were reversely transcribed using the M-MLV reverse transcriptase kit or miRNA reverse transcriptase kit following the accompanying instructions. SYBR Green Premix Ex Taq™ II was mixed with cDNA and specific primers for qRT-PCR assay on a CFX96 TM real-time PCR detection system (Bio-Rad Laboratories, Hercules, USA). Relative gene expressions were calculated using the 2^−ΔΔ*C*t^ method with GAPDH as an internal control for mRNAs and circRNAs, and U6 as an internal control for miRNAs [27, 29].

### 2.10. Dual-Luciferase Evaluation

Cells cotransfected with hsa-miR-217 mimics or negative control (miR-NC), psiCHECK-2/hsa_circ_0004543 3′-UTR (WT), or psiCHECK-2/hsa_circ_0004543 3′-UTR mutated (MT) plasmid were used for hsa_circ_0004543 activity analysis, while cotransfected with psiCHECK-2/HIF-1Α 3′-UTR (WT) or psiCHECK-2/HIF-1Α 3′-UTR mutated (MT) plasmid were used for HIF-1Α activity analysis using Lipofectamine 3000. The dual-luciferase reporter assay kit was then used following the manufacturer's procedures [26, 29].

### 2.11. RNA Pull-Down Evaluation

GenePharma (Shanghai, China) provided biotin-labeled hsa_circ_0004543 probes (hsa_circ_0004543) and negative controls (oligoes). PierceTM Magnetic RNA-Protein Pull-Down kit was used for RNA pull-down assay. Briefly, the miRNA binding to hsa_circ_0004543 was determined by qRT-PCR after it was enriched by incubating streptavidin agarose magnetic beads with biotin-labeled hsa_circ_0004543 probes or negative control first, and then with the cell lysates from SiHa or C-4I cells [30].

### 2.12. Western Blotting Investigation

Total protein was extracted with RIPA lysis buffer. Proteins (25 *μ*g) were used for target protein expression determination based on separation on an 8% SDS-PAGE gel, followed by the transfer on a polyvinylidene fluoride (PVDF) membrane, incubating at 4°C overnight in primary and secondary antibodies for 1 h at RT, and developing with SuperSignal West Dura Extended Duration Substrate after it was washed three times with TBST [26, 27].

### 2.13. Statistical Assay

SPSS 19.0 (IBM, SPSS, Chicago, US) was used. Tests were conducted with one-way analysis of variance followed by Tukey's post hoc test for multiple groups and Student's *t*-test for two groups. Associations between gene expressions were evaluated with Pearson's correlation assay. *P* < 0.05 was statistically significant.

## 3. Results

The purpose of the current work was to explore the role and the ceRNA mechanism of hsa_circ_0004543 in regulating CC oncogenesis and metastasis. Based on the bioinformatics analysis and literature review, we hypothesized that hsa_circ_0004543 expression was upregulated in CC cells and tissues, which contributed to increased CC cell viability, colony proliferation, migration, and invasion. It also inhibited cell apoptosis by regulating the hsa-miR-217/HIF-1*α* axis. Hsa_circ_0004543 levels in human CC tissues and cells were determined with qRT-PCR. Direct binding between hsa-miR-217 and hsa_circ_0004543 or HIF-1a was predicted by the interactome or TargetScan and was verified with a dual-luciferase reporter gene assay with or without RNA pull-down. Aggressive phenotypes of CC cells including cell viability, colony proliferation, apoptosis, migration, and invasion were detected with the CCK-8 assay, colony formation assay, flow cytometry assay, and transwell assay, respectively. The mechanism of hsa_circ_0004543 in CC development was further assessed by silencing hsa_circ_0004543 with/without hsa-miR-217 silencing or HIF-1a overexpression. Associations between gene expressions were evaluated with Pearson's correlation analysis.

### 3.1. hsa_circ_0004543 Is Upregulated in CC Tissues and Cells

**Figure 1:**
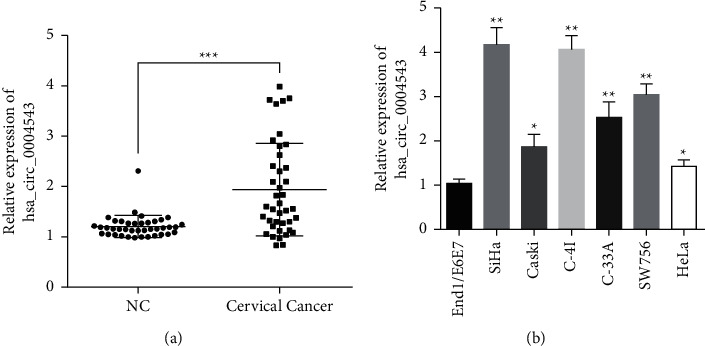
hsa_circ_0004543 was amplified in cervical cancer tissue and cells. hsa_circ_0004543 expression was determined with quantitative reverse transcriptase polymerase chain reaction (qRT-PCR). (a) The hsa_circ_0004543 expression was considerably upregulated in CC patient tissues compared with paired paracancerous normal tissues (NC) (*n* = 40). (b) The hsa_circ_0004543 expression was considerably upregulated in CC cells (SiHa, CaSki, C-4I, C-33A, SW756, and HeLa) compared with End1/E6E7 (normal human cervical epithelial cells) (^*∗*^*p* *<* 0.05, ^*∗∗*^*p* < 0.01, and ^*∗∗∗*^*p* < 0.001).

To explore the function of hsa_circ_0004543 in CC oncogenesis and progress, we first collected paired CC and self-matched negative control (NC) paracancerous tissues from 40 CC patients, followed by the analysis of the hsa_circ_0004543 expression by qRT-PCR. The data proved that hsa_circ_0004543 expression was extremely higher in CC tissues versus NC tissues (Figure 1(a)). We also analyzed the hsa_circ_0004543 level in CC cells, and the hsa_circ_0004543 level was significantly higher in CC cells (SiHa, CaSki, C-4I, C-33A, SW756, and HeLa) versus End1/E6E7 cells (Figure 1(b)).

### 3.2. hsa_circ_0004543 Stimulates CC Growth and Metastasis

**Figure 2:**
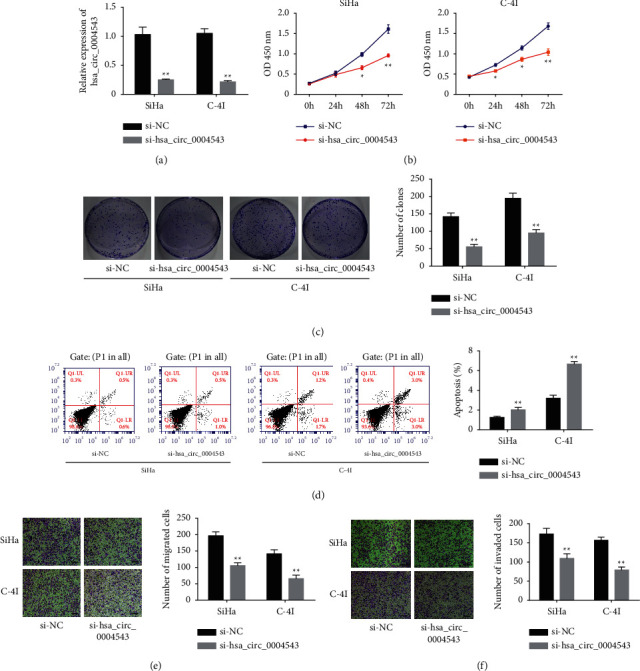
hsa_circ_0004543 silencing inhibited viability, colony formation, migration and invasion, and prompted apoptosis in CC cells. (a) The qRT-PCR assay validated successful hsa_circ_0004543 silencing with si-hsa_circ_0004543 in SiHa and C-4I cells. (b) Viability, (c) colony formation ability, (d) apoptosis, (e) migration capacity, and (f) invasion capacity of SiHa and C-4I cells after they were transfected with si-NC or si-hsa_circ_0004543 (^*∗*^*p* *<* 0.05 and ^*∗∗*^*p* < 0.01).

To explore the effects of hsa_circ_0004543 on CC malignant phenotypes, silencing RNA (siRNA) specifically targeting hsa_circ_0004543 (si-hsa_circ_0004543) was respectively transfected to two CC cells (SiHa and C-4I) and yielded the highest hsa_circ_0004543 expression for hsa_circ_0004543 knockdown. The results showed that transfecting si-hsa_circ_0004543 in SiHa and C-4I cells produced a significantly reduced hsa_circ_0004543 expression (Figure 2(a)). Viabilities of SiHa and C-4I cells at 0, 24, 48, and 72 h after transfecting si-NC or si-hsa_circ_0004543 were determined with the CCK-8 test. This revealed that hsa_circ_0004543 silencing significantly decreased the viability (OD value) of both SiHa and C-4I cells (Figure 2(b)). The colony proliferation abilities of SiHa and C-4I cells were evaluated with the colony formation assay, which revealed that hsa_circ_0004543 silencing significantly repressed the colony proliferation abilities of both the SiHa and C-4I cells (Figure 2(c)). In the meantime, flow cytometric assays confirmed that apoptosis rates were considerably amplified in SiHa and C-4I cells after hsa_circ_0004543 silencing (Figure 2(d)). Moreover, invasion and migration capabilities of SiHa and C-4I cells were evaluated with a transwell assay, which indicated that hsa_circ_0004543 knockdown significantly repressed CC cell migration (Figure 2(e)) and invasiveness (Figure 2(f)) capabilities.

### 3.3. hsa_circ_0004543 Sponges hsa-miR-217 in CC Cells to Increase HIF-1*α* Expression

**Figure 3:**
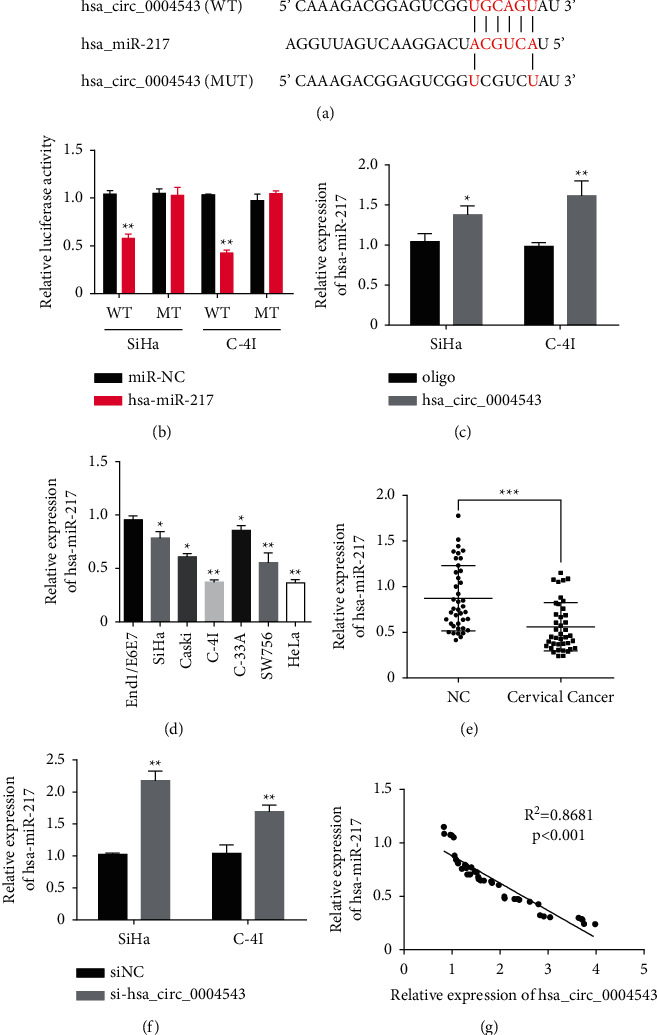
hsa_circ_0004543 was identified to serve as a sponge for hsa-miR-217 in CC cells. (a) Diagram of potential binding sites between hsa-miR-217 and hsa_circ_0004543 (https://circinteractome.nia.nih.gov/) with mutation sites for specific binding assay. (b) Dual-luciferase reporter activity of SiHa and C-4I cells cotransfected by hsa_circ_0004543 3′UTR wild-type or mutated reporter with or without hsa-miR-217 mimics. (c) hsa-miR-217 was pulled down and enriched with biotin-labeled hsa-miR-217 specific probe in CC cell lysates. (d) hsa-miR-217 expression in human CC cells (SiHa, CaSki, C-4I, C-33A, SW756, and HeLa) was significantly downregulated compared with normal human cervical epithelial cells (End1/E6E7). (e) hsa-miR-217 expressions in paired CC and paracancerous (NC) tissues from 40 local CC patients detected by qRT-PCR. (f) hsa-miR-217 expressions in hsa_circ_0004543 silenced SiHa and C-4I cells determined with qRT-PCR. (g) Pearson's correlation analysis of associations between hsa-miR-217 and hsa_circ_0004543 expressions in CC patient tissues (*n* = 40) (^*∗*^*p* *<* 0.05, ^*∗∗*^*p* < 0.01, and ^*∗∗∗*^*p* < 0.001).

To explore the molecular mechanism of hsa_circ_0004543 in CC carcinogenesis and development, the potential miRNAs which had complementary binding sites with hsa_circ_0004543 were first predicted based on the use of the Circular RNA Interactome (https://circinteractome.nia.nih.gov/) (Figure 3(a)). This analysis was followed in SiHa and C-4I cells by dual-luciferase reporter activity analysis and RNA pull-down assays, respectively. The luciferase activity of the wild-type hsa_circ_0004543 3′UTR reporter gene in SiHa and C-4I cells was significantly inhibited when hsa-miR-217 was overexpressed with mimics, while the inhibited luciferase activity of the hsa_circ_0004543 3′UTR reporter gene was rescued when the predicted binding sites of hsa_circ_0004543 3′UTR with hsa-miR-217 was mutated (Figure 3(b)). The interaction between hsa_circ_0004543 and hsa-miR-217 in SiHa and C-4I cells was further confirmed by the RNA pulldown, which showed that the hsa_circ_0004543 probe (hsa_circ_0004543) could pulldown more hsa-miR-217 than control oligoes (Figure 3(c)). Moreover, the relationship between hsa_circ_0004543 and hsa-miR-217 was explored based on the determination of the hsa-miR-217 level in CC cells and tissues by qRT-PCR. The data confirmed that the hsa-miR-217 expression was meaningfully decreased in CC cells (SiHa, CaSki, C-4I, C-33A, SW756, and HeLa) compared to normal human cervical epithelial cells (Figure 3(d)) and was considerably declined in CC tissues compared to paired paracancerous normal tissues from 40 local CC patients (Figure 3(e)). Meanwhile, significantly upregulated hsa-miR-217 expression was found in SiHa and C-4I cells after the hsa_circ_0004543 was silenced (Figure 3(f)). Associations between hsa_circ_0004543 and hsa-miR-217 expressions were evaluated further using Pearson's correlation coefficient, which showed that hsa_circ_0004543 level was significantly negatively related to the hsa-miR-217 level in CC tissues from 40 CC patients (Figure 3(g)).

**Figure 4:**
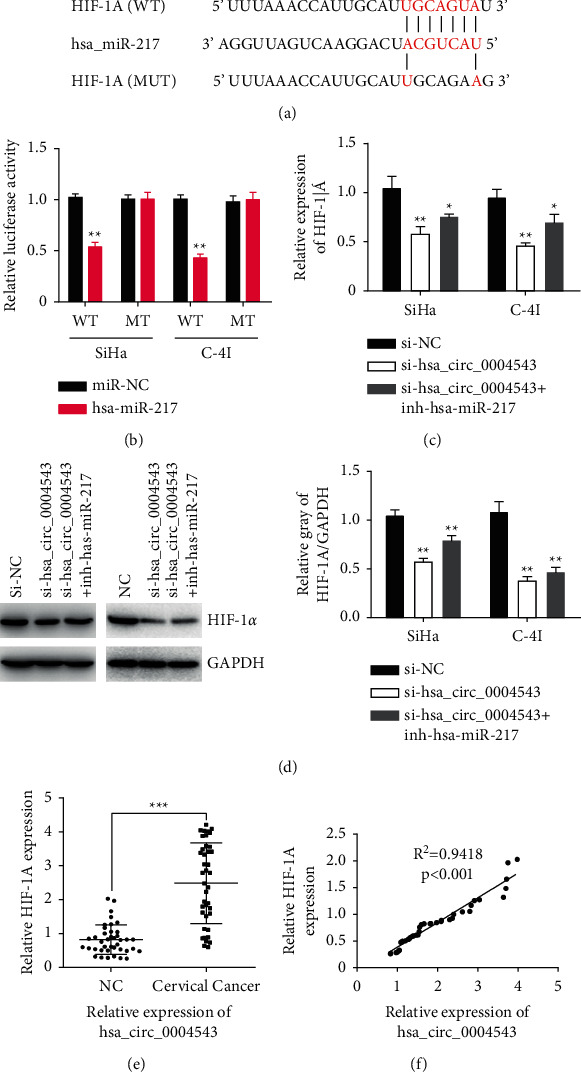
hsa_circ_0004543 increased the hypoxia-inducible factor (HIF-1*α*) expression by sponging hsa-miR-217 in CC. (a) Diagram of potential binding sites between hsa-miR-217 with HIF-1a (http://www.targetscan.org/) and mutation sites for the specific binding assay. (b) Dual-luciferase activity of SiHa and C-4I cells cotransfected by HIF-1a 3′UTR wild-type (WT) or mutated (MT) reporter with or without hsa-miR-217 mimics. (c) HIF-1a messenger ribonucleic acid (mRNA) expressions in SiHa and C-4I cells after hsa_circ_0004543 silencing (si-hsa_circ_0004543) with or without hsa-miR-217 inhibitor (inh-hsa-miR-217) determined using qRT-PCR. (d) HIF-1*α* protein expression of SiHa and C-4I cells after hsa_circ_0004543 silencing with or without inh-hsa-miR-217 determined using western blot. (e) Relative HIF-1a expressions in paired CC and paracancerous (NC) tissues determined with qRT-PCR (*n* = 40). (f) Association between HIF-1a and hsa_circ_0004543 expressions in CC patient tissues investigated by Pearson's correlation analysis (*n* = 40) (^*∗*^*p* *<* 0.05, ^*∗∗*^*p* < 0.01, and ^*∗∗∗*^*p* < 0.001).

To reveal the ceRNA network trigger by hsa_circ_0004543, potential mRNAs which had complementary binding sites with hsa-miR-217 were first predicted with the online tool TargetScan (http://www.targetscan.org/) (Figure 4(a)), followed by the dual-luciferase reporter activity assay in SiHa and C-4I cells, respectively. The luciferase activity of the wild-type HIF-1a 3′UTR reporter gene was considerably inhibited when hsa-miR-217 was overexpressed using mimics in SiHa and C-4I cells, while inhibited luciferase activity of the HIF-1a 3′UTR reporter gene was rescued when predicted binding sites of HIF-1a 3′UTR with hsa-miR-217 were mutated (Figure 4(b)). Moreover, we investigated whether hsa_circ_0004543 sponges hsa-miR-217 to control HIF-1*α* level, and HIF-1a expression in CC cells was first detected with qRT-PCR. The data showed that hsa_circ_0004543 silencing in CC cells inhibited HIF-1*α* expression considerably at both mRNA (Figure 4(c)) and protein (Figure 4(d)) levels, which was partly reversed by the cotransfection of the hsa-miR-217 inhibitor (inh-hsa-miR-217) (Figures 4(c) and 4(d)). Furthermore, the expression of HIF-1a was measured by qRT-PCR in 40 CC patient tissues, which showed that HIF-1a expression was significantly amplified in CC tissues compared with paracancerous normal tissues (Figure 4(c)). Moreover, the association between hsa_circ_0004543 and HIF-1a expressions in 40 CC patient tissues was analyzed using Pearson's correlation analysis. This revealed that the hsa_circ_0004543 level was considerably positively related to HIF-1a expression (Figure 4(d)). These findings suggested that hsa_circ_0004543 stimulated CC oncogenesis and development via sponging hsa-miR-217 induced the upregulation of HIF-1*α*.

### 3.4. The hsa-miR-217/hsa-miR-217/HIF-1*α* Axis Promotes CC Cell Malignant Phenotypes

**Figure 5:**
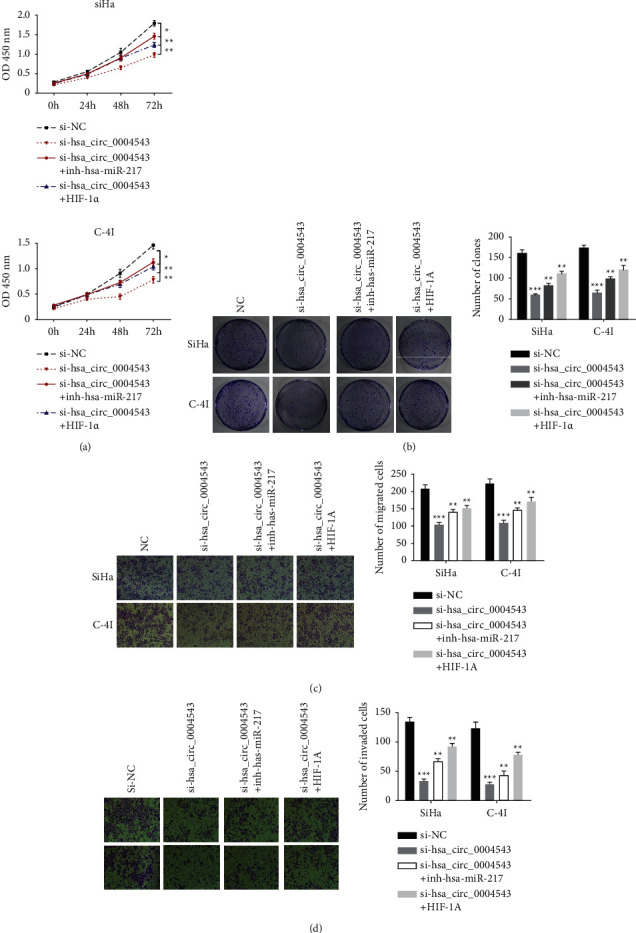
hsa-miR-217 knockdown or HIF-1a overexpression rescued hsa_circ_0004543 silencing inhibited malignant phenotypes in CC cells. (a) Viability of SiHa and C-4I cells after hsa_circ_0004543 silencing with/without inh-hsa-miR-217 or HIF-1a overexpression determined using the CCK-8 assay. (b) Colony proliferation abilities of SiHa and C-4I cells after hsa_circ_0004543 silencing with/without inh-hsa-miR-217 or HIF-1*α* overexpression determined using colony formation assay. (c) Cell migration capability of SiHa and C-4I cells after hsa_circ_0004543 knockdown with/without inh-hsa-miR-217 or HIF-1a overexpression detected by transwell assay without Matrigel. (d) Cell invasion capability of SiHa and C-4I cells after hsa_circ_0004543 silencing with/without inh-hsa-miR-217 or HIF-1*α* overexpression detected using transwell assay with Matrigel (^*∗*^*p* *<* 0.05; ^*∗∗*^*p* < 0.01, and ^*∗∗∗*^*p* < 0.001).

After being cotransfected using si-NC, si-hsa_circ_0004543, si-hsa_circ_0004543 + inh-hsa-miR-217, or si-hsa_circ_0004543+HIF-1a, viabilities of SiHa and C-4I cells were first determined based on the use of the CCK-8 assay, and the data validated that hsa_circ_0004543 silencing time-dependently decreased OD value (0–72 h) and was partly rescued by hsa-miR-217 silencing or HIF-1a overexpression (Figure 5(a)). Colony proliferation abilities of SiHa and C-4I cells after hsa_circ_0004543 silencing with/without inh-hsa-miR-217 or HIF-1a overexpression were detected by the colony formation assay, which showed that hsa_circ_0004543 silencing in SiHa and C-4I cells inhibited colony proliferation abilities (Figure 5(b)), and this inhibitory efficacy was partly rescued by hsa-miR-217 knockdown or HIF-1a overexpression. Finally, cell migration and invasiveness capacities in SiHa and C-4I cells were investigated based on the transwell assay without or with Matrigel. The data showed that hsa_circ_0004543 silencing inhibited both migration (Figure 5(c)) and invasiveness (Figure 5(d)) capabilities, which was partly reversed by hsa-miR-217 knockdown or HIF-1a overexpression. These findings indicated that hsa-miR-217 knockdown or HIF-1a overexpression reversed hsa_circ_0004543 silencing and inhibited malignant phenotypes in CC cells.

## 4. Discussion

circRNAs are noncoding RNAs that are highly stable in eukaryotic cells. Hsa_circ_0004543 has been newly identified as a significantly upregulated circRNA in CC tissues by circRNA microarray [12]. Nevertheless, its function and clinical implication in malignancy are still unknown. Therefore, we performed a comprehensive investigation of hsa_circ_0004543 in CC oncogenesis and its progression. In this work, we first confirmed a considerably higher hsa_circ_0004543 expression in CC tissues versus paired paracancerous normal tissues from 40 local CC patients, as well as in six different CC cell lines compared with normal human cervical epithelial cells, indicating the potential oncogenic role of hsa_circ_0004543 in CC. Secondly, we revealed that hsa_circ_0004543 silencing in CC cells significantly inhibited cell viability, colony proliferation, migration and invasiveness, and induced apoptosis.

Accumulating evidence has shown that transcriptional regulation between ncRNAs and mRNAs plays an essential function in CC cancer progression, including growth, migration, invasion, and multidrug resistance. As an important class of ncRNAs, miRNAs also play a critical role in regulating cell functions via degradation of target genes, therefore regulating cell proliferation, apoptosis, and metastasis. circRNA may be competitive endogenous RNA (ceRNA) for miRNAs to regulate expression of downstream targets of mRNAs, and the ceRNA networks are important mechanisms to elucidate the posttranscriptional regulation in CC [26, 31–35]. Therefore, we explored further the ceRNA network mediated by hsa_circ_0004543 in the current study.

We used the online tool Circular RNA Interactome which predicted miRNAs harboring complementary binding sequences with hsa_circ_0004543 and identified the potential candidate of hsa-miR-217. The direct binding between hsa_circ_0004543 and hsa-miR-217 was further confirmed with dual-luciferase reporter activity examination in SiHa and C-4I cells. Meanwhile, hsa-miR-217 was identified to be inhibited in both CC cell lines and tissues, indicating the oncosuppressor role of hsa-miR-217 in CC. Moreover, hsa_circ_0004543 silencing in SiHa and C-4I cells considerably upregulated hsa-miR-217 expression, and Pearson's correlation assay discovered a negative association between hsa-miR-217 with hsa_circ_0004543 expressions in 40 CC patient tissues. These findings suggested that hsa_circ_0004543 directly interacted with hsa-miR-217 to promote the aggressive phenotypes of CC cells.

We used the online tool TargetScan to predict the mRNAs harboring complementary binding sequences with hsa-miR-217 and identified the potential candidate of HIF1A. The direct binding between HIF1A and hsa-miR-217 was further confirmed with a dual-luciferase reporter activity assay in SiHa and C-4I cells. Hypoxia is a typical representative of middle-late stage solid cancers and plays a key role in promoting malignancy cells to adapt to the hypoxia microenvironment in cancer by regulating the HIF transcriptional factors [36]. HIF-1*α*, encoded by HIF1A, is a commonly expressed HIF*α* isoform in various cells and the most essential regulator of oxygen homeostasis [37]. HIF-1*α* changes are associated with the outcomes of patients with various cancers, suggesting its critical role in carcinogenesis [38]. Our advanced investigation revealed that HIF-1a mRNA levels were considerably increased in CC tissues and were positively associated with hsa_circ_0004543. Meanwhile, knockdown of hsa_circ_0004543 considerably inhibited the expression of HIF-1*α* at both the mRNA and protein levels, and led to reduced viability and colony proliferation ability in CC cells. These effects were partly reversed by inhibiting hsa-miR-217 or overexpressing HIF-1a. Outcomes indicated that HIF-1a directly interacted with hsa-miR-217 via the sponging activity of hsa_circ_0004543 to stimulate CC progression. Hence, hsa_circ_0004543 may function as a ceRNA of hsa-miR-217 to inhibit HIF-1*α* degradation and thus stimulate the growth and metastasis of CC cells.

In conclusion, our current work showed that the hsa_circ_0004543 level was significantly amplified in CC patients and cells that promoted viability, colony proliferation, migration and invasiveness, and repressed apoptosis by sponging hsa-miR-217 to upregulate the HIF-1*α* level in CC cells. These changes thus contributed to the CC cell's oncogenesis and progression. Our results emphasized the potential function of hsa_circ_0004543 as a newly identified oncogenic ncRNA and that targeting the hsa_circ_0004543/hsa-miR-217/HIF-1*α* axis may provide a new therapeutic strategy to treat CC.

## Data Availability

The data used to support the findings of this study are included within the article.

## Additional Points

(1) circ_0004543 is an oncogenic factor in CC. (2) circ_0004543 acts as a ceRNA of hsa-miR-217 to upregulate HIF-1*α*. (3) Targeting the hsa_circ_0004543/hsa-miR-217/HIF-1*α* axis is a potential management strategy for CC.

## References

[1] Collaborators G. B. D. S. C. (2020). The global, regional, and national burden of stomach cancer in 195 countries, 1990-2017: a systematic analysis for the Global Burden of Disease study 2017. *Lancet Gastroenterol Hepatol*.

[2] Sung H., Ferlay J., Siegel R. L., Laversanne M., Soerjomataram I., Jemal A. (2021). Global cancer statistics 2020: GLOBOCAN estimates of incidence and mortality worldwide for 36 cancers in 185 countries. *CA: A Cancer Journal for Clinicians*.

[3] Allemani C., Weir H. K., Carreira H. (2015). Global surveillance of cancer survival 1995-2009: analysis of individual data for 25 676 887 patients from 279 population-based registries in 67 countries (CONCORD-2). *The Lancet*.

[4] Stapley S., Hamilton W. (2011). Gynaecological symptoms reported by young women: examining the potential for earlier diagnosis of cervical cancer. *Family Practice*.

[5] Lim A. W., Ramirez A. J., Hamilton W., Sasieni P., Patnick J., Forbes L. J. (2014). Delays in diagnosis of young females with symptomatic cervical cancer in England: an interview-based study. *British Journal of General Practice*.

[6] Cohen P. A., Jhingran A., Oaknin A., Denny L. (2019). Cervical cancer. *The Lancet*.

[7] Gong J., Jiang H., Shu C. (2019). Integrated analysis of circular RNA-associated ceRNA network in cervical cancer. *Medicine*.

[8] Salzman J., Chen R. E., Olsen M. N., Wang P. L., Brown P. O. (2013). Cell-type specific features of circular RNA expression. *PLoS Genetics*.

[9] Jeck W. R., Sorrentino J. A., Wang K. (2013). Circular RNAs are abundant, conserved, and associated with ALU repeats. *RNA*.

[10] Memczak S., Jens M., Elefsinioti A. (2013). Circular RNAs are a large class of animal RNAs with regulatory potency. *Nature*.

[11] Li Z., Huang C., Bao C. (2015). Exon-intron circular RNAs regulate transcription in the nucleus. *Nature Structural & Molecular Biology*.

[12] Gao Y.-L., Zhang M.-Y., Xu B. (2017). Circular RNA expression profiles reveal that hsa_circ_0018289 is up-regulated in cervical cancer and promotes the tumorigenesis. *Oncotarget*.

[13] Zhang J., Zhao X., Zhang J., Zheng X., Li F. (2018). Circular RNA hsa_circ_0023404 exerts an oncogenic role in cervical cancer through regulating miR-136/TFCP2/YAP pathway. *Biochemical and Biophysical Research Communications*.

[14] Cai H., Zhang P., Xu M., Yan L., Liu N., Wu X. (2019). Circular RNA hsa_circ_0000263 participates in cervical cancer development by regulating target gene of miR‐150‐5p. *Journal of Cellular Physiology*.

[15] Song T., Xu A., Zhang Z. (2019). CircRNA hsa_circRNA_101996 increases cervical cancer proliferation and invasion through activating TPX2 expression by restraining miR‐8075. *Journal of Cellular Physiology*.

[16] Li H., Jia Y., Wang Y. (2019). Targeting HIF-1alpha signaling pathway for gastric cancer treatment. *Pharmazie*.

[17] Wei T.-T., Lin Y.-T., Tang S.-P. (2020). Metabolic targeting of HIF-1*α* potentiates the therapeutic efficacy of oxaliplatin in colorectal cancer. *Oncogene*.

[18] Zhang L., Huang S.-T., Feng Y.-L. (2017). The bidirectional regulation between MYL5 and HIF-1*α* promotes cervical carcinoma metastasis. *Theranostics*.

[19] Bertout J. A., Patel S. A., Simon M. C. (2008). The impact of O2 availability on human cancer. *Nature Reviews Cancer*.

[20] Rankin E. B., Giaccia A. J. (2016). Hypoxic control of metastasis. *Science*.

[21] Lu X., Kang Y. (2010). Hypoxia and hypoxia-inducible factors: master regulators of metastasis. *Clinical Cancer Research*.

[22] Carmeliet P., Jain R. K. (2011). Molecular mechanisms and clinical applications of angiogenesis. *Nature*.

[23] Semenza G. L. (2012). Hypoxia-inducible factors in physiology and medicine. *Cell*.

[24] Balamurugan K. (2016). HIF-1 at the crossroads of hypoxia, inflammation, and cancer. *International Journal of Cancer*.

[25] Hong Q., Li O., Zheng W. (2017). LncRNA HOTAIR regulates HIF-1*α*/AXL signaling through inhibition of miR-217 in renal cell carcinoma. *Cell Death & Disease*.

[26] Gu Q., Hou W., Shi L., Liu H., Zhu Z., Ye W. (2021). Circular RNA ZNF609 functions as a competing endogenous RNA in regulating E2F transcription factor 6 through competitively binding to microRNA-197-3p to promote the progression of cervical cancer progression. *Bioengineered*.

[27] Hu S., Chang J., Ruan H. (2021). Cantharidin inhibits osteosarcoma proliferation and metastasis by directly targeting miR-214-3p/DKK3 axis to inactivate *β*-catenin nuclear translocation and LEF1 translation. *International Journal of Biological Sciences*.

[28] O’Leary V. B., Smida J., Matjanovski M. (2017). The circRNA interactome-innovative hallmarks of the intra- and extracellular radiation response. *Oncotarget*.

[29] Jia J., Ouyang Z., Wang M. (2021). MicroRNA-361-5p slows down gliomas development through regulating UBR5 to elevate ATMIN protein expression. *Cell Death & Disease*.

[30] Cen J., Liang Y., Huang Y. (2021). Circular RNA circSDHC serves as a sponge for miR-127-3p to promote the proliferation and metastasis of renal cell carcinoma via the CDKN3/E2F1 axis. *Molecular Cancer*.

[31] Ma X., Zhang Q., Du J., Tang J., Tan B. (2021). Integrated analysis of ceRNA regulatory network associated with tumor stage in cervical cancer. *Frontiers in Genetics*.

[32] Wang T., Zhang X.-D., Hua K.-Q. (2021). A ceRNA network of BBOX1-AS1-hsa-miR-125b-5p/hsa-miR-125a-5p-CDKN2A shows prognostic value in cervical cancer. *Taiwanese Journal of Obstetrics & Gynecology*.

[33] Ding L., Zhang H. (2019). Circ-ATP8A2 promotes cell proliferation and invasion as a ceRNA to target EGFR by sponging miR-433 in cervical cancer. *Gene*.

[34] Wang Y., Wang L., Wang W., Guo X. (2020). Overexpression of circular RNA hsa_circ_0001038 promotes cervical cancer cell progression by acting as a ceRNA for miR-337-3p to regulate cyclin-M3 and metastasis-associated in colon cancer 1 expression. *Gene*.

[35] Liu S., Li B., Li Y., Song H. (2021). Circular RNA circ_0000228 promotes the malignancy of cervical cancer via microRNA-195-5p/lysyl oxidase-like protein 2 axis. *Bioengineered*.

[36] Semenza G. L. (2007). Hypoxia-inducible factor 1 (HIF-1) pathway. *Science’s STKE : Signal Transduction Knowledge Environment*.

[37] Semenza G. L. (2004). Hydroxylation of HIF-1: oxygen sensing at the molecular level. *Physiology*.

[38] Yang W., Ma J., Zhou W. (2019). Reciprocal regulations between miRNAs and HIF-1*α* in human cancers. *Cellular and Molecular Life Sciences*.

